# Impact of intraoperative methadone on early extubation and opioid utilization in intravenous drug users undergoing cardiac surgery

**DOI:** 10.1186/s12871-025-03446-8

**Published:** 2025-12-23

**Authors:** Abigail Hoff, Matthew B. Ellison, Galen Kabulski, Alec Statler, Conner D. Funke, J. W. Awori Hayanga, Pavithra R. Ellison

**Affiliations:** 1 West Virginia University School of Medicine, Morgantown, WV 26501 United States of America; 2 Department of Pharmacy, West Virginia University School of Medicine, Morgantown, WV United States of America; 3 Department of Anesthesiology, West Virginia University School of Medicine, Morgantown, WV United States of America; 4 Department of Cardiovascular and Thoracic Surgery, West Virginia University, Morgantown, WV United States of America; 5 Division of Cardiovascular and Thoracic Anesthesiology, West Virginia University, Morgantown, United States of America; 6 University of Cincinnati Medical Center, Cincinnati, OH United States of America

**Keywords:** Methadone, Early extubation, Pain control, Opioid use, Intravenous drug users, Early extubation after cardiac surgery

## Abstract

**Objectives:**

To evaluate the impact of intraoperative methadone on early extubation and opioid use in the perioperative period (intraoperative, <12 hours postoperatively, >12 hours postoperatively) in intravenous drug users (IVDU) undergoing cardiac valvular surgery.

**Methods:**

Retrospective chart review of the electronic medical record. Data was collected from a single large rural academic medical center over a 14-month period. Patients with active or history of IV drug use who underwent cardiothoracic surgery and who received methadone intraoperatively were included in the analysis. Exclusion criteria included discharge less than 72 hours postoperatively, a documented allergy to methadone, and patients who were on oral methadone preoperatively. 61 patients received a single dose of IV methadone intraoperatively. 24 matched comparator patients did not receive any methadone. These 2 groups were compared on time to extubation using a Cochran-Mantel-Haenszel method to analyze the categorical data (intraoperative, <12 hours postoperatively, >12 hours postoperatively). Furthermore, groups were compared based on morphine equivalents intraoperatively and postoperatively, and the amount of anesthesia given intraoperatively and postoperatively by examining dexmedetomidine (intraoperative and postoperative) and ketamine requirements using Independent sample t-tests.

**Measurements and main results:**

91% of patients in the methadone group were extubated within 12 hours compared to 66% in the control group (p = 0.0006) demonstrating a significant linear association. There was no significant difference with regard to use of morphine equivalents, however, there was a significant difference in the dose of dexmedetomidine administered postoperatively between the methadone and control group (42.9 vs. 108, p = 0.039).

**Conclusions:**

Administering a single dose of IV methadone intraoperatively in patients with an IV drug use history may facilitate earlier extubation and reduce postoperative sedation (dexmedetomidine) requirements.

## Introduction

Acute postoperative pain is a common complication in surgical patients, particularly those that have undergone cardiac surgery [1]. Multiple factors contribute to this severe pain including sternotomy, chest tubes, intravascular cannulations, rib fractures, surgical incisions, and breathing tube placement [1]. Typically, pain is worse during the first two days postoperatively and medical teams rely heavily on the use of intravenous (IV) opioids [2]. Nevertheless, many patients still report their pain to be moderate to severe during this time despite intravenous and supplemental IV bolus opioid therapy [3]. 

It is well documented that uncontrolled pain may lead to adverse clinical consequences and impair recovery in these patients. Puntillo and colleagues evaluated pain scores and subsequent clinical events in 74 patients undergoing cardiac and abdominal vascular surgery [4]. They reported that patients with higher pain intensity had a significantly greater incidence of postoperative atelectasis [4]. Additionally, Roediger et al. reported that sympathetic nervous system activation secondary to uncontrolled pain may induce myocardial ischemia and arrhythmias [5]. 

Most commonly, short-acting opioids are used to treat this pain, and may be administered via a variety of methods. Some patients receive continuous infusions of short-acting opioids, others receive bolus dosing, and still others self-administer with a patient-controlled analgesia (PCA) device. Repeated bolus dosing of short-acting analgesics can result in fluctuating levels of pain control for the patient [1]. Severe post-operative pain may fluctuate in nature with higher doses being required for analgesia one hour and then incurring the risk of respiratory depression in future hours. High-quality quality effective and safe pain control may only occur intermittently.

An alternative approach to intermittent bolus dosing of short-acting analgesics is the administration of a single dose of methadone. Methadone is a long-acting opioid with a half-life of 24 to 36 h when administered at large doses of 20 to 30 mg making for optimal pharmacokinetics for a medication for patients with substance abuse disorders [6, 7].

Murphy et al. analyzed the effects of a single intraoperative dose of methadone versus fentanyl on pain scores and morphine requirements after cardiac surgery. A single dose of methadone (0.3 mg/kg) or fentanyl (12 mcg/kg) was given to patients undergoing cardiac surgery with cardiopulmonary bypass. Postoperative analgesic requirements and pain scores were assessed at 2, 4, 8, 12, 24, 48, and 72 h after tracheal extubation. Postoperative morphine requirements were reduced from a mean of 10 mg in the fentanyl group to 6 mg in the methadone group (*P* < 0.001).^1^ Pain scores were significantly lower in the methadone group at every time interval except 4 h [1]. Additionally, the incidence of opioid-related adverse events was not increased in patients that received methadone.

At our institution, there is a high incidence of patients with a history of IV drug use (IVDU) that develop endocarditis requiring cardiac valvular surgery. Because of their history of drug abuse, these patients are tolerant to standard doses of opioids utilized in the immediate postoperative period [8]. Postoperative pain management commonly necessitates higher and more frequent dosing of pain medications in these patients. A single dose of methadone given intraoperatively has been shown to provide superior pain control in the general cardiac surgery patient population but it is unknown whether this same benefit exists in those with a history of IVDU.

Early extubation after cardiac surgery has been shown to decrease postoperative morbidity and mortality and adequate pain control is essential in order to achieve this [9]. Acceptable pain control is crucial to maintain stable hemodynamic parameters and facilitate early transition from the intensive care unit (ICU) to the floor setting. In addition to decreasing morbidity and mortality, early extubation has additional benefits. The earlier patients are extubated, the sooner they can ambulate, thus decreasing length of stay and overall hospital costs [10, 11]. Additionally, it has been proposed that earlier extubation may decrease a patient’s risk for developing pneumonia [12]. In a 1999 study published by Coplin and colleagues, it was concluded that extubation delay was associated with a statistically significant increase in the risk of pneumonia. Early extubation can contribute to less atelectasis and thus decrease the risks for ventilator-associated pneumonia.

The purpose of this study is to evaluate whether the intraoperative use of methadone has an impact on early extubation and opioid utilization in the first 12 h after cardiac valvular surgery in IVDU patients.

## Methods

### Patients

Patients aged 18 years or older were included in the study if they had a documented history of IV drug use, underwent a cardiothoracic surgical procedure, and received a dose of methadone intraoperatively at West Virginia University Medicine – J.W. Memorial Hospital within the study period of 05/2018 to 06/2019. IV drug use was identified retrospectively as patients with a positive urine drug screen in the past 6 months and self-reported history. Patients were excluded if they were discharged less than 72 h postoperatively, had a documented allergy to methadone, or were taking oral methadone prior to surgery. Institutional review board approval was obtained for this study.

### Study design

This study was designed as a retrospective chart review of patients who received an intraoperative dose of methadone during a cardiothoracic procedure over this 14-month period. Patients were identified through the use of the Epic electronic medical record. Methadone doses were rounded to the nearest 5 mg at the discretion of the anesthesia provider. The dose of methadone was administered post-induction and prior to sternotomy.

The primary endpoint was time to extubation which was defined as: intraoperatively, less than 12 h postoperatively, and greater than 12 h postoperatively. Secondary endpoints included opioid utilization intraoperatively and postoperatively, as well as analgesic requirements intraoperatively and postoperatively. Opioid utilization was defined as morphine milligram equivalents (MME) of all opioids administered to the patient. Opioid conversions to morphine equivalents were assessed using the CDC Guideline for Prescribing Opioids for Chronic Pain [13]. Patients received a variety of opioids including fentanyl, hydromorphone, oxycodone, morphine, and hydrocodone. Additional anesthesia adjuncts were defined as dexmedetomidine and ketamine use in micrograms and milligrams respectively intraoperatively and postoperatively where appropriate.

### Statistical approach

We examined the association between groups (patients who received intraoperative methadone versus patients who did not receive intraoperative methadone) on time of extubation, which was categorized as extubation intraoperatively, within 12 h postoperatively, and more than 12 h postoperatively. We accounted for the ordinal nature of categorical time of extubation and utilized Cochran-Mantel-Haenszel methods for analysis.

We compared groups on morphine equivalents intraoperatively and postoperatively, and the amount of anesthesia adjuncts given intraoperatively and postoperatively by examining dexmedetomidine (intraoperative and postoperative) and ketamine requirements using Independent sample t-tests.

All statistical analyses were performed using SAS 9.4 (SAS Institute, Cary NC) and statistical significance was determined as *p* < 0.05.

## Results

A total of 61 patients met the inclusion criteria of IVDU and received a single IV dose of methadone intraoperatively while undergoing a cardiothoracic procedure. Twenty-four IVDU matched cohort patients were identified and served as a comparator group. Baseline demographics identified by the patient’s actual body weight, gender, and surgical type are depicted in Table 1.. Surgical type was divided into groups based on the valvular operation. If a patient received two or more valve repairs or replacements in a single operation, they were classified as multiple. There were no differences between groups. Patients in the methadone arm had an average body weight of 75.5 kg while patients in the control group had an average weight of 77.5 kg. Approximately 55% of the patients in the overall study were female. The average dose of intraoperative methadone administered was 0.261 mg/kg, given as a single intravenous bolus.

**Table 1.** Baseline demographics

	**Methadone (n = 61)**	**Control (n = 24)**
Average weight (kg)	75.5	77.5
Female, n (%)	33 (52)	13 (54)
Methadone dose (mg)	18.9	-
Methadone dose per kg (mg/kg)	0.261	-
Surgical type, n		
Aortic	5	4
Mitral	6	5
Tricuspid	43	10
Multiple	6	3
Other	1	2

Methadone dose stratified by patient weight is seen in Table 2. Fifty-one patients (84%) received a flat dose of 20 mg. A linear correlation was described with patients having a larger baseline weight receiving a larger one-time dose of methadone. Patients who received a one-time 10 mg dose of methadone had a lower average mg/kg dose of methadone at 0.142 mg/kg compared to an average of 0.286 mg/kg in the other groups.

**Table 2:** Methadone dose by weight

Methadone dose (mg)	Number of Patients	Average weight (kg)	Methadone dose per kg (mg/kg)
10	8	72.2	0.142
20	51	75.2	0.280
25	1	86.2	0.290
30	1	103.9	0.289

A significant linear association between group and time of extubation was observed, QS = 11.8146, *p* = 0.0006 (Table 3). Twenty-eight patients in the methadone group were extubated in the operating room (OR), 25 were extubated within 12 h, and five were extubated greater than 12 h compared to the 3, 11, and 8 patients respectively in the control group. Thus, there was sufficient evidence to suggest that a greater proportion of those individuals who received intraoperative methadone were extubated earlier than those individuals who did not receive intraoperative methadone.

**Table 3:** Group by time to extubation

Variable	Extubated in OR	Extubated < 12 h	Extubated > 12 h	Test Statistic	*p*-value
Methadone, n (%)	28 (48.28)	25 (43.10)	5 (8.62)	11.8146	0.0006
Control, n (%)	3 (13.64)	11 (50.00)	8 (36.36)		

There was no significant difference between the two groups with respect to the use of morphine equivalents (Table 4). In the intraoperative setting, morphine equivalents in the methadone were 126.2 compared to 151.8 in the control group (*p* = 0.136). In the postoperative setting, morphine equivalents were 27.5 compared to 38.2 in the control group (*p* = 0.06). Similarly, there was no significant difference between groups in the amount of adjunct anesthetics given intraoperatively evaluated as ketamine and dexmedetomidine utilization (Table 5). The mean milligrams of ketamine given intraoperatively were 34.6 compared to 35.4 in the control group (*p* = 0.917). The mean micrograms of dexmedetomidine given intraoperatively were 17.9 in the methadone group compared to 10.5 in the control group (*p* = 0.160).

However, there was a significant difference in the amount of dexmedetomidine given postoperatively between the two groups. In the methadone group, the mean micrograms of dexmedetomidine given were 42.9 compared to 108 in the control group (*p* = 0.039). Those individuals who received intraoperative methadone received significantly less dexmedetomidine postoperatively compared to those individuals who did not receive methadone intraoperatively (Table 5).

**Table 4:** Group by morphine milligram equivalents

Variable	Methadone (*n* = 61)	Control (*n* = 24)	Test Statistic	*p*-value
Intraoperative	126.2	151.8	1.54	0.136
Postoperative	27.5	38.2	1.99	0.06

**Table 5:** Adjunct anesthetic requirements

Variable	Methadone (*n* = 61)	Control (*n* = 24)	Test Statistic	*p*-value
Intraoperative - ketamine (mg)	34.65	35.45	0.10	0.917
Intraoperative – dexmedetomidine (mcg)	17.93	10.55	−1.42	0.160
Postoperative – dexmedetomidine (mcg)	42.98	108	2.10	0.039

## Discussion

Postoperative pain and delayed extubation-related sequelae are common complications in cardiac surgical patients. Unfortunately, most patients still report their pain to be moderate to severe in the first several days after surgery despite intravenous and supplemental IV bolus opioid therapy. Intraoperative and early extubation after cardiac surgery has been shown to decrease postoperative morbidity and mortality [9, 14]. Adequate pain control is essential to achieve this outcome.

In the current study, the authors found that administering a single dose of IV methadone intraoperatively in patients with a history of IVDU facilitated intraoperative and early extubation and decreased postoperative dexmedetomidine sedation requirements. Dexmedetomidine is frequently utilized for postoperative sedation in the cardiothoracic intensive care units. In this study, dexmedetomidine was titrated at a rate of 0.2 mcg/kg/hr to the Richmond Agitation Sedation Scale of 0 to −2. In patients who received methadone intraoperatively, only 8% were extubated greater than 12 h after surgery, compared to 36% of patients in the control group. It is well documented that earlier time to extubation is one of the best predictors for postoperative morbidity and mortality [9, 14]. In a 2009 study, Camp and colleagues examined 2735 patients; 1164 patients underwent early extubation (< 6 h after surgery) and 1571 patients had a conventional extubation (>6 h after surgery). The authors concluded extubation within 9 h was the best predictor of improved postoperative morbidity and mortality [9]. Additionally, early extubation was a predictor of prolonged survival up to 16 months [9]. These findings highlight the overall clinical significance of earlier extubation seen after receiving a single dose of intraoperative methadone in addition to statistical significance.

Decreased postoperative dexmedetomidine requirement is also a clinically meaningful outcome well established in scientific literature. Dexmedetomidine is a selective central alpha-2 receptor agonist that works as a sedative and anesthetic. Although typically well tolerated, this medication may lead to hypotension bradycardia in post-operative cardiac patients. The Clinical Practice Guidelines for the Prevention and Management of Pain, Agitation/Sedation, Delirium, Immobility, and Sleep in the ICU (PADIS) conclude that sedatives including dexmedetomidine may predispose patients to increased mortality [15, 16]. Additionally, The ability to decrease postoperative dexmedetomidine use post cardiac surgery for patients in an ICU setting is clinically significant and meaningful.

While opioid utilization in morphine equivalents was not statistically different between the two groups, patients in this study received a lower average dose of methadone than was studied in primary literature. Murphy et al. saw a statistically significant decrease in pain scores after administration of a single bolus dose of 0.3 mg/kg of methadone in patients with normal pain tolerance. In our analysis, patients with an IV drug use history received an average dose of 0.261 mg/kg methadone. While our results did not demonstrate a statistically significant decline in opioid use, this may be due to the fact that our patients received a lower dose of methadone. In our cardiac intensive care unit, all post-operative cardiac patients are placed on a standard scheduled opioid regimen. We believe that this may have led to the similarity in post-op opioid usage. It is hypothesized that the doses used were likely insufficient, and a dose greater than 0.3 mg/kg may be needed for patients actively using and abusing opioids.

Although this study was conducted in patients who had a history of IVDU, it is hypothesized that these results could be replicated in patients who are opioid-tolerant at baseline from chronic opioid use not related to IV drug abuse. With a growing number of patients on chronic opioid therapy and de-sensitized mu receptors, these findings may be pertinent to consider in a broader group of patients throughout the perioperative setting. A single dose of IV methadone administered intraoperatively can provide pain relief for up to 1 to 2 days postoperatively and could be especially useful in patients who are opioid-tolerant with difficult-to-manage pain, no matter the mechanism.

The study has several limitations. First, it is a single-center retrospective review with a small sample size. Second, the routine clinical use of intraoperative methadone in cardiothoracic surgery began in 2018, limiting the sample size. Third, if patients were admitted while taking outpatient opioids or were transferred directly to the OR from inpatient status, their ongoing use of opioids might have influenced their postoperative requirements. Lastly, the optimal intraoperative dose of methadone as seen in previous literature was not achieved in our study population.

While this study did not achieve the optimal intraoperative dose of methadone, it did demonstrate that methadone is a reasonable option for IVDU patients as demonstrated by an earlier time to extubation and decreased postoperative anesthesia requirements. This is the first study in the literature to evaluate such an outcome in patients with a history of IVDU undergoing a cardiac valvular operation.

## Data Availability

The datasets used and/or analysed during the current study are available from the corresponding author on reasonable request.

## Abbreviations

IVDU: Intravenous Drug Use
IV: Intravenous
PCA: Patient Controlled Analgesia
ICU: Intensive Care Unit
OR: Operating Room
MME: Morphine Milligram Equivalent
